# The Effects of Functional Electrical Stimulation of Hip Abductor and Tibialis Anterior Muscles on Standing and Gait Characteristics in Patients with Stroke

**DOI:** 10.3390/jcm14072309

**Published:** 2025-03-28

**Authors:** Sami S. AlAbdulwahab, Abdulaziz S. Aldhaferi, Abdulrahman M. Alsubiheen, Sultan H. Alharbi, Fahad H. Alotaibi, Mohammed A. Alghamdi, Abdulrahman Basonbul, Atta El Sousai, Mohammed M. Al-Harbi, Muneera M. Almurdi

**Affiliations:** 1 Rehabilitation Health Sciences Department, College of Applied Medical Sciences, King Saud University, Riyadh 11433, Saudi Arabia; swahab@ksu.edu.sa (S.S.A.); aalsubiheen@ksu.edu.sa (A.M.A.); 2 Physiotherabia Clinics, Riyadh 12215, Saudi Arabia; asdhaferi@gmail.com; 3 King Fahad Medical City, Rehabilitation Hospital, Riyadh 12231, Saudi Arabia; sbhalharbi@kfmc.med.sa (S.H.A.); pt-fahad@hotmail.com (F.H.A.); mabdualghamdi@kfmc.med.sa (M.A.A.); abasonbul@kfmc.med.sa (A.B.); 4 Kingdom Hospital and Consultant Clinic, Riyadh 13316, Saudi Arabia; attaelsousai@yahoo.com; 5 Department of Family and Community Medicine, College of Medicine, King Saud University, Riyadh 11433, Saudi Arabia; dr.alzogibi@gmail.com

**Keywords:** stroke, functional electrical stimulation, gait, spasticity, hip abductor muscles, tibialis anterior muscles

## Abstract

**Background/Objectives:** Functional electrical stimulation (FES) has been used to improve the quality of life of patients with stroke. Rehabilitation programs focus on standing and walking, which are vital to functional independence and keystone ingredients in functional competency. To examine the effects of simultaneous continuous ongoing FES of gluteus medius (GMed) and tibialis anterior (TA) muscles at isometric contraction during standing and walking in patients with stroke. **Methods:** Short- and long-term FES management programs of GMed and TA muscles during different conditions have been used in patients with stroke. FES was applied to hip abductors and dorsiflexor muscles of the affected limb during four different conditions: passive hip abduction and ankle dorsiflexion, respectively (condition 1), sit-to-stand (condition 2), 10 m walk test (condition 3), and walking on C-mill treadmill (condition 4). The Modified Ashworth Scale (MAS), Five Times sit-to-stand test (FTSST), 10-m walk test (10-MWT), and C-mill treadmill were used to assess spasticity in the hip adductor and calf muscles, sit-to-stand performance, and temporal–spatial characteristics, respectively. **Results**: Short- and long-term FES management programs significantly reduced spasticity in the hip adductor and calf muscles and improved sit-to-stand performance, gait speed, and gait temporal–spatial characteristics. **Conclusions:** Short- and long-term FES management programs of GMed and TA muscles can quickly and effectively improve the spasticity and ambulation of patients with stroke. Further research incorporating gait analysis with randomized controlled samples is needed.

## 1. Introduction

Stroke is the second leading cause of death and the third leading cause of disability worldwide. Stroke is linked with aging, poor diet, and sedentary life, and has become a leading cause of adult acquired motor disability, which imposes a significant burden on survivors, their careers, and the community. Fifteen million people experience strokes worldwide each year. Of these, five million die, and another five million are permanently disabled [1]. In the Middle East, the prevalence of stroke has risen significantly in the last decade, exceeding that of developed countries [2] and become one of the fastest-growing cerebrovascular diseases in the Kingdom of Saudi Arabia (KSA), leading to increased morbidity and mortality, thus raising the social and economic burden [3,4,5].

Stroke has many clinical features and deficits, such as muscle weakness, altered muscle tone, abnormal movement patterns, and limited range of motion [6]. However, the most common clinical features/deficits after stroke are movement difficulty and functional disability. Almost 80% of patients who have experienced stroke have hemiparesis or weakness of the involved side with abnormal spastic synergetic movement patterns, and impairing daily living activities, including standing and gait ability [1]. Such muscle weakness and abnormal spastic synergetic movements in the involved side have been reported to encourage patients with stroke to adopt different abnormal standing and gait patterns, with obvious impaired kinematics and kinetics of lower limb, e.g., a dropped or equinius foot, decreased/loss of knee and hip flexion during the swing phase, lateral trunk instability, and difficulty in shifting weight to the involved side during the stance phase [7,8,9]. These impairments cause short stride length, decreased push force at late stance, reduced walking speed, reduced walking endurance, balance deficit [10,11], and increased energy expenditure while walking [12], lowering stroke patients’ independence and limiting their social participation [13]. Therefore, an intensive rehabilitation program is crucial to improve impaired kinematics and kinetics and facilitate a better quality of life for patients who have experienced stroke.

Previous studies have examined a variety of rehabilitation techniques, including robotic-assisted therapy [14], task-specific training [15], and functional electrical stimulation (FES) [16,17], to address standing and gait deficits after stroke. FES of the hip abductor and anterior tibialis muscles is used as preparation for walking or during walking [18,19,20]. Hip abductor muscles, particularly the gluteus medius (GMed), operate as pelvic stabilizers during one-legged standing and throughout the stance phase of locomotion. They also act as pelvic rotators, rotating the opposing side of the pelvis forward and making the swing phase of the gait more energy efficient. As a result, these muscles play a crucial role in standing, gait, and hip biomechanics [21]. On the other hand, the ankle dorsiflexor muscles, especially the tibialis anterior (TA), have an important role in the gait cycle. Total or partial paresis to this muscle causes a drop in the foot in patients with stroke. During the swing phase, this makes ground clearance difficult, leading to inefficient gait compensations such as circumduction and hip hiking.

Although FES of the hip abductor and anterior tibialis are reported to improve the gait of patients with stroke, further studies are still needed. The limited number of participants in FES studies, unclear FES parameters used, and preliminary assessment tools of efficiency of FES [19,20,22] are common problems associated with FES application in patients with stroke, confirming the need for further studies on the use of FES to improve standing and walking. Clinically, it is already known that standing and walking are the prime objectives of rehabilitation programs because of their vital role in functional independence, and they are keystone ingredients in functional competency [23]. Therefore, the objectives of this study were to examine the effects of simultaneous continuous ongoing FES of the GMed and TA muscles on spasticity in hip adductor and calf muscles, as well as on sit-to-stand ability and gait parameters in patients with stroke.

## 2. Materials and Methods

### 2.1. Study Design

A repeated measures design was used across sessions in this study.

### 2.2. Study Setting and Ethical Approval

This study was conducted in the rehabilitation department at King Fahad Medical City, Riyadh, Kingdom of Saudi Arabia. It was ethically approved by the IRB at King Fahad Medical City (No. 21-562). A brief oral description of the study objectives and experimental procedures was provided for each patient. Afterward, a written consent form was obtained from each patient. 

### 2.3. Patients, Sampling Method, Recruitment, and Data Collection

This study included a convenience sample of patients with chronic stroke. Their inclusion criteria were a first-time stroke event, being cognitively intact, and having the ability to walk independently with/without assistance aids for more than 3 min. Patients with orthopedic disorders, surgery on the lower extremities, and psychological or emotional problems were excluded from this study.

### 2.4. Sample Size Calculation

The sample size estimation was determined using G-power with a large effect size of η2 = 0.14\η2 = 0.14, statistical power of 0.90, an alpha level 0.05, repeated measure ANOVA test, and one group. The required sample size was N = 12–15.

## 3. Instruments

### 3.1. Modified Ashworth Scale (MAS)

The MAS is one of the most widely used clinical evaluation methods for measuring spasticity. The MAS scale is a six-point ordinal scale for grading resistance during passive muscular stretching. Spasticity is graded as follows: 0 = normal muscle tone; 1 = slight increase in muscle tone; 2 = slight increase in muscle tone (less than half) of the ROM; 3 = more marked increase in muscle tone, but limb easily moved; 4 = considerable increase in muscle tone, with passive movement difficult; and 5 = limb rigid in flexion or extension. The MAS is a reliable and valid scale to measure spasticity in patients with stroke [24].

### 3.2. Five Times Sit-to-Stand Test (FTSST)

The FTSST is frequently used to assess balance and standing ability in patients with stroke. The subject is instructed to sit on a comfortable chair with their arms crossed on their chest, then stand up upright and sit down five times, as fast as possible. The start time of the entire test is recorded when the clinician says “Go” and is the end time is when the subject’s buttocks touch the chair for the fifth repetition. Between repetitions of the test, the clinician instructs the subject to stand up fully and asks them not to touch the chair’s back throughout each repetition. A stopwatch records the time taken to complete the test in seconds. The FTSST is valid, reliable, and easy to perform [25].

### 3.3. 10-Meter Walk Test (10 MWT)

The 10-MWT can be used for determining gait velocity. Subjects are timed as they walk normally along a 10-m walkway. A total distance of 10 m is measured and the start and end 2 m points are marked with tape. The subject is instructed to walk at their comfortable speed for 10 m from the ‘start’ sign. This test is a method for calculating the time required to walk a 10 m distance, minus the start and end of 2 m for acceleration and deceleration. A stopwatch records the time taken to complete the test in seconds. The 10 MWT is a reliable and valid method to assess the gait speed of patients with stroke [26,27,28].

### 3.4. C-Mill Analysis System

This is a treadmill with a highly sensible single force embedded platform (C-Mill, Motek Force link, Calembour, The Netherlands), used to analyze the temporal and spatial elements of the gait. The C-Mill consists of a single large uniaxial force plate (0.8 × 3.0 m), with a safety harness (body support system) and programs to collect information about many temporal–spatial parameters during walking. It is a reliable and valid method to measure the temporospatial parameters of patients with stroke [29,30].

## 4. Functional Electrical Stimulation Management Programs

### 4.1. Functional Electrical Stimulation (FES) Unit

The FES unit was a two-channel, small, portable, battery-powered device (Digital Dual-Channel TENS unit Comfy stim). The unit was set to operate in constant mode with a frequency of 35 Hz, pulse width of 80 μsec, and intensity causing visible and minimal continuous isometric contraction of both the gluteus medius and tibialis anterior muscles of the affected limb via four self-adhesive electrodes; each electrode is square in shape and measures 5.1 cm × 5.1 cm. These stimulation parameters were standardized for all patients, with the exception of the intensity, which was adjusted to ensure visible isometric contraction.

### 4.2. FES Management Programs

There were two FES management programs. The first FES management program was a short-term protocol of ongoing and simultaneous FES of hip abductors and dorsiflexor muscles of the affected limb during four different conditions, which were passive hip abduction and passive ankle dorsiflexion, respectively (condition 1), sit-to-stand five times (condition 2), 10 m walk test (condition 3), and walking for 3–5 min on the C-mill treadmill (condition 4). It was carried out before the regular prescribed medical rehabilitation program.

The second FES management program was a long-term home protocol of ongoing and simultaneous FES of hip abductors and dorsiflexor muscles of the affected limb during walking for 15 min, during three sessions per day with at least 3 h intervals between each session, for one week. Patients’ carers had been trained to deliver the second program safely at home.

## 5. Assessment Procedure

### Baseline Assessment Procedure

Spasticity in the hip adductor and plantar flexor muscles, sit-to-stand ability, 10-m walking ability, and temporospatial gait parameters of each patient were assessed twice on two consecutive days to establish baseline performance for each patient. A 2-min rest between each test was permitted. The baseline serves as a reference point for monitoring and evaluating the effects of FES management programs on patients’ physical performance. It provides a clear indication of a patient’s current situation and ability that is used to accurately measure the effectiveness of the FES management programs.

Patients were assessed on the first day, then re-assessed on the second day using the MAS, FTSST, 10 MWT, and C-mill treadmill, at least 1 h before the time when they were due to take any drugs prescribed for spasticity. The baseline assessment steps were as follows:

The spasticity in the hip adductor muscle was determined by passive hip abduction movement from the natural anatomical position to 45 degrees within 5 s. This procedure was repeated three times at 30-s intervals, and the average grading resistance to passive movement using the modified Ashworth scale was recorded. Spasticity in the calf muscle was determined by passive dorsiflexion movement from maximum plantarflexion to maximum available dorsiflexion within 5 s. This procedure was repeated three times at 30-s intervals, and the average grading resistance to passive movement was also recorded.

For sit-to-stand ability, the patient was asked to sit on the edge of a comfortable chair with their hip and knee in approximately 90 degrees of flexion, and their feet just touching the force platform of the C-Mill treadmill. Afterward, the patient was asked, on the command of “off you go”, to perform the sit-to-stand test as fast as possible five times, and the time taken to do this was recorded using a digital stopwatch.

The 10-m walking performance was recorded by instructing the patient to sit on a comfortable chair and then asking them to stand up and walk at a comfortable speed for 10 m. The time taken to complete the test was measured by a digital stopwatch.

**Scheme 1:**
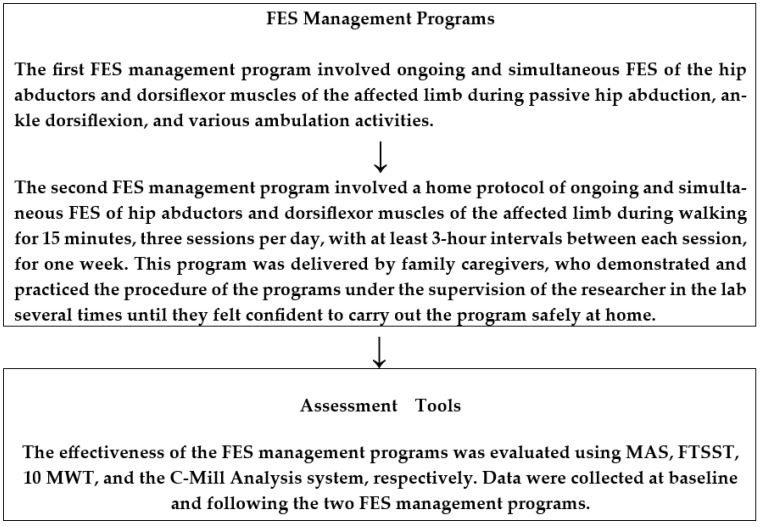
Simple illustration of the experimental management and assessment protocol.

Walking temporospatial parameters baseline measurement was recorded by helping the patient to sit on a comfortable chair, secured with suspended harnesses, and instructing them to stand on the C-Mill treadmill. The patient was asked to walk while the speed of the C-Mill treadmill was gently and gradually increased until it reached the patient’s comfortable speed. Then, the patient was asked to walk at an average self-selected walking speed for 3–5 min. Then, the gait parameters were analyzed (as shown in Scheme 1).

## 6. Patient Preparation for FES Management Programs

**Figure 1:**
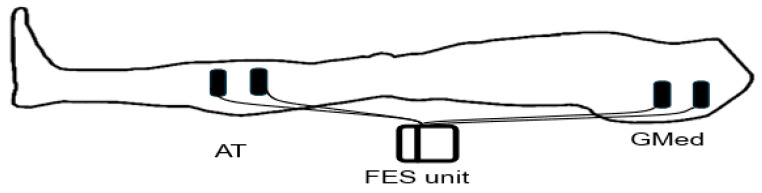
A simple illustration showing the placement of FES unit electrodes on the GMed and TA muscles of the affected side.

Patients wearing short, comfortable pants were instructed to relax in a supine position on a comfortable table. The skin over the GMed and TA areas of the affected side was cleaned and moistened with alcohol swabs to reduce possible skin resistance. Then, the FES unit electrodes were attached to the GMed and TA of the affected side. For the GMed muscle, the electrodes were placed on a triangle between the iliac tubercle, the posterior superior iliac spine, and the greater trochanter. This triangle is easily identified by palpation during minimum resistance to active hip abduction. For the TA, electrodes were placed on the point approximately 1/4 to 1/3 of the way between the knee joint and the ankle joint in the lateral parallel direction of the medial shaft of the tibia. The FES unit active electrode was placed over the belly of the GMed and TA, while the other electrode was placed 3 cm below the active electrode in the longitudinal axis. Then, electrodes were connected to the FES unit (Figure 1).

## 7. Short-Term FES Management Program and Assessment Procedure

Condition 1. FES of the hip GMed and TA muscles were delivered while the patient was lying in a supine position on a comfortable table A graduate increase in the intensity of FES was applied until the patient felt only a tingling sensation, which lasted for 15 s. Afterward, a further graduate increase in the intensity was applied until obvious and comfortable isometric contraction of the GMed and TA muscles was shown, and this was maintained for 2 min. Next, simultaneously with ongoing FES of the GMed and TA muscles, a passive hip abduction and ankle dorsiflexion were carried out throughout the available range of motion. This procedure was repeated three times, with a 30-s interval, and the average of the grading resistance to passive movement on the modified Ashworth scale was recorded.

Condition 2. The FES unit was turned off, and the patient was asked and helped to sit on a comfortable chair with the FES unit tightened on his/her waist. Afterward, the FES unit was turned on, and the intensity was gradually increased until obvious and comfortable isometric contraction of both the GMed and TA muscles was noticed. Then, the patient was asked to perform the sit-to-stand test as fast as possible, five times, and the time taken to do this was recorded. Then, the patient was asked to sit on the chair, and the FES unit was turned off. The patient was asked to relax for 2 min.

Condition 3. With the patient sitting, the FES unit was turned on, and the intensity was gradually increased until obvious and comfortable isometric contraction of both GMed and TA muscles was noticed. Then, the patient was asked to stand up, take deep breaths, and was instructed on the command “Go” to perform the 10-m walk test five times; the time taken to do this was recorded. Then, the patient was asked to relax for 2 min.

Condition 4. The patient was asked to stand on the C-Mill treadmill and was secured with suspended harnesses. The FES unit was turned on, and the intensity was gradually increased until obvious and comfortable isometric contraction of both the GMed and TA muscles was noticed. Then, the C-Mill treadmill was turned on; the speed was gradually increased while the patient was waking until it reached the patient’s comfortable speed. After this, the patient was asked to walk at an average self-selected walking speed for 3–5 min while the simultaneous FES of both the GMed and TA muscles was being conducted. Then, the gait parameters were recorded (Scheme 1).

## 8. Long-Term FES Management Program and Assessment Procedure

At the end of the short-term FES management program, operational instructions, application, demonstration, and precautions of the FES were explained to the carers to prepare them for conducting this program safely at home. Then, the following instructions were given to the carers: 1. Help the patient to put on short pants, 2. Sit the patient on a comfortable chair; 3. Moisten the skin over the red and black square marks with alcohol swabs, 4. Place the self-adhesive electrodes over the square marks, ensuring that the black wire electrodes (negative electrodes) are placed over the black squares and the red wire electrodes (positive electrodes) are placed over the red squares, 5. Turn on the FES unit and gradually increase the intensity until the patient reports a tingling sensation. Stay at this intensity for about 1 min, then gradually increase the intensity until you see an obvious contraction in the hip and leg muscle, 6. Attach the FES unit to the patient’s waist belt and ask him/her to stand up and walk around for 15 min, ensuring you stay around him/her throughout the session, 7. After 15 min, ask the patient to sit again, turn off the FES unit, and disconnect the electrodes, 8. Repeat the above procedures three times a day for 1 wk, with a period of no less than 3 h between sessions.

The carers procedure for the programs was demonstrated to carers and they practiced under the supervision of the researcher in the lab several times until they felt confident to carry out the program safely. The carers were asked to contact the researcher whenever necessary.

The next day after completing this FES management program, the degree of spasticity, sit-to-stand, and walking ability were again assessed, without and then during ongoing FES application, exactly as described earlier in the baseline assessment procedure section and short-term management program procedure section (Scheme 1).

## 9. Statistical Analysis

All statistical data were analyzed using SPSS software (version 20.0). The means and standard deviations were calculated to show the descriptive statistics of the dependent variables. Repeated measures, one-way ANOVA and the LSD test were used for multiple comparisons. The statistical significance level was α = 0.05.

## 10. Results

A total of 14 male patients with chronic stroke, with a mean age of 55.93 years, BMI of 27.76 kg/m^2^, and post-stroke duration of 7.5 ± 3 months (at the chronic stage, ranging from 6–10 months) were included in this study. Ten of them had the left side affected, while four had the right side affected.

### 10.1. Baseline Data

The mean ± SD of MAS scores of the ankle plantar flexor and hip adductor muscles, FTSST, 10 MWT and C-Mill treadmill force plate results of gait parameters, including the stride length, step length, and step width that were measured on day 1 and day 2 were comparable, with no statistically significant differences (*p* > 0.05) (Table 1).

### 10.2. Short-and-Long-Term Management Programs

The MAS score of spasticity in the ankle plantar flexor and hip adductors muscles, FTSST, and 10 MWT speed time were significantly reduced in the short- and long-term management programs compared to the baseline results, and also demonstrated further significant reduction at the long-term management program with FES (Table 2). Moreover, stride length, step length and step width were significantly increased in the short- and long-term management programs compared to the baseline scores, similarly with further significant increase at the long-term management program with FES (Table 2).

The result of this study showed that both the short- and long-term management programs of TA and GM improve spasticity, FTSST, 10 MWT, stride, step length, and step width in patients with stroke. Moreover, this improvement was better with using FES during the long-term management program.

## 11. Discussion

This study investigated the influence of ongoing continuous FES of the GMed and TA muscles during walking on muscle tone, sit-to-stand ability, walking ability, and spatiotemporal gait parameters. It showed the feasibility and benefit of applying FES simultaneously to the GMed and TA muscles during walking as a fast, immediate, effective physical rehabilitation management program to improve the gait performance and spasticity in patients with stroke.

FES has been widely used to manage motor control of patients with stroke. It reduces spasticity, increases muscle strength, and activates motor units [18,19,20]. It increases the activity of the cerebral sensory–motor cortex, improves functional movement, and is used as a motor learning tool to improve the physical activity of patients with stroke [31]. In this context. the short- and long-term FES management programs used in this study improve spasticity, gait performance, and sit-to-stand ability. These results are in agreement with the previous FES application studies [18,19,20].

Muscle weakness of the involved lower limb is considered the main troublesome impairment in patients with stroke [32]. It makes standing and walking difficult and unsafe [33]. Weakness in the GMed contributes to decreased walking speed and limited control of lateral stability during the stance phase. Moreover, weakness in TA strength is reported to impair control of ankle dorsiflexion in the swing phase and cause a decrease in walking speed, increased time for foot clearance during the swing phase, and increased double limb support duration [34,35]

It appears that such muscle weakness, with its associated difficulties in walking, can be managed within a short period of time by our short- and long-term FES programs, which instantly improved the gait parameters and sit-to-stand ability during ongoing FES to the gluteus medius and TA of patients with stroke. This improvement could be due to the additional motor unit activation and strength of the hip abductor and TA muscles, which in turn stabilize the hip and adjust the foot position, respectively. It has been reported that the stronger the paretic hip abductors of patients with stroke, the faster the performance of the timed up-and-go test, the higher the ambulation ability, the better the adjustment of the center of mass, and the faster the stair climbing [36]. Moreover, FES of the TA muscle combined with the moving treadmill significantly improves the foot gait of patients with stroke and enhances the normalization of hip flexion and knee flexion [37]. Thus, it can be suggested that the short-term FES management program recruited and depolarized more motor units of the disused muscle fibers, further increased the below-threshold nerve/muscle action potentials, or increased the frequency of motor units firing, that facilitated contractile strength in the GMed and TA muscles.

The activation of additional motor units will increase the number of active muscle fibers and subsequently generate stronger muscle contraction [33]. Thus, the short-term FES management program may activate more motor units that produce strong functional GMed and TA muscle contractions. Furthermore, the frequency of motor unit firing rates is another possible explanation for the instant improvement of gait parameters and sit-to-stand ability during ongoing FES to the GMed and TA of patients with stroke. A gradual increase in muscle contraction is associated with a gradual and smooth increased firing rate of the motor unit until the maximum rate is reached, and vice versa [32]. With a continuous ongoing FES program during walking and sit-to-stand, the firing rates of motor units’ action potentials may be generated and modulated based on the combination of both the patient’s capacity to activate the GMed and TA muscles and additional activation initiated by FES.

Moreover, the Type 1 and type 2 muscle fibers within the GMed and TA muscles may be activated collectively and voluntarily by patients and electrically induced by FES. The Type 1 muscle fibers may be activated by patients because they have a low voluntary activation threshold in the weakened disused muscle. Meanwhile, the Type 2 muscle fibers may be selectively activated by FES because they have large diameter motor nerves with low resistance to FES. Typically, Type 1 muscle fibers with low activation thresholds are recruited first. If they cannot generate the required force for the specific activity, the Type 2 muscle fibers are engaged. Therefore, this might happen with the short-term FES management program, as FES has been reported to stimulate Type 2 muscle fibers before Type 1 muscle fibers [38].

In another context, spasticity frequently interferes with the motor function of patients with stroke. Spasticity in the calf muscle of patients with stroke causes a foot drop, disrupting walking and balance [38]. The short-term management program reduced spasticity in the calf and hip adductor muscles, as measured by the modified Ashworth scale. Such reduction could be due to proper facilitation of reciprocal inhibition due to continuous ongoing FES to TA and hip abductor muscles of the paretic side. The FES-induced muscle contractions activate reciprocal inhibition through the disynaptic Ia inhibitory pathway, similar to voluntary contraction. Upon stimulation of an agonist muscle (e.g., TA and GMed in this study), signals quickly travel to the spinal cord. Within the spinal cord, Ia afferent fibers synapse with inhibitory interneurons, thereby decreasing the excitability of the antagonist muscle (e.g., ankle plantar flexors and hip adductors in this study) [39,40,41]. This robust mechanism ensures that activation of the agonist muscle results in the inhibition of the antagonist, thereby ensuring coordinated movement and delivering significant therapeutic benefits, such as diminished spasticity and minimized unwanted co-contraction [42]. It has been reported that controlling the abnormal extensor synergetic spastic pattern in the lower limb improves standing and walking ability [43]. The reduction in TA and GMed muscle spasticity and the improvement of gait parameters and sit-to-stand of our study support these reports.

Regarding comparison with similar studies that used FES to TA and GMed muscles during walking with a foot switch to turn the electrical stimulators on-off, Cho et al. (2015) reported improvements of 15.9% in the stride length and 46.6% in gait speed [34]. Their program comprised 30 minute sessions, five times per week for four weeks. Further, Araki et al. (2020) demonstrated a 5.3% improvement in gait speed and 12.3% in stride length with foot-on-off FES to AT and GMed muscles during walking [19]. On the other hand, Chung et al. (2014) showed a 28.9% improvement in stride length after a program of 30 min sessions, five times a week for six weeks [44]. Sabut et al. (2010) showed an improvement of 26.28% in gait speed, 21.27% in step length, and 20.41% in stride length after using FES program sessions of 60 min, five days a week for 12 weeks [45]. Likewise, both FES programs in the present study also led to a significant improvement in the gait parameters, which were far better than those of the previous studies over a short period. The short-term FES program yielded an improvement of 37.9% in gait speed, 30.5% in step length, and 28.8% in stride length during simultaneous, ongoing FES to TA and GMed muscles during walking. The long-term FES program demonstrated an improvement of 33.6% in gait speed, 41.6% in step length, and 36.5% in stride length after a home program of 15 min sessions, three times a day for seven days.

Such remarkable improvement could be due to simultaneous, continuous, ongoing FES to TA and GMed muscles at visible isometric contraction during walking, which may control agonist–antagonist co-contraction between the TA/calf muscles and hip abductor/hip adductor muscle through reciprocal inhibition activation. The reduction in spasticity in the plantar flexor and hip adductor muscles, as measured by the MAS, during simultaneous, continuous, ongoing FES to TA and GMed muscles, could support the assumption of controlled agonist–antagonist co-contraction.

It is also worth mentioning that the long-term FES management program is manageable by patients and caregivers. All patients completed the long-term FES management of 15 min of walking three times a day for a week, as described in the present study, with no complaints of skin irritation or muscle aches. They felt comfortable and happy with it and reported the benefits of FES in improving their standing gait abilities.

Overall, this study’s findings highlight the potential of FES as a valuable adjunctive approach in the comprehensive rehabilitation of stroke patients, offering immediate improvements in gait performance, muscle strength, and spasticity management.

Although the present study showed significant improvement in the degree of spasticity, sit-to-stand, and gait performance, some limitations are worth mentioning. Temporospatial parameters were only measured to determine gait improvement. However, the 3D gait analysis system is worth using to measure kinetic and kinematic gait parameters. The study sample was convenient, with limited post-stroke variation; further study is needed with a bigger randomized sample that covers a wide range of post-stroke stages. Due to difficulties in recruiting patients, the present study does not include a control group, which limits the ability to compare the results with those of other interventions. However, the baseline readings recorded in the present study improve the accuracy and interpretability, enabling within-subject comparisons to measure true changes from the intervention while accounting for individual variability. This enhances internal validity by distinguishing intervention effects from natural fluctuations. Additionally, the study has not evaluated the long-term benefits of FES after the management program has been discontinued, which could be explored in a future study.

## 12. Conclusions

It can be concluded that continuous ongoing FES to TA and Gmed of the affected side of patients with stroke is a highly beneficial technology for the reduction of spasticity, improvement of gait parameters, and sit-to-stand ability for patients with stroke. It may be considered more feasible, applicable, and effective than other programs.

## Figures and Tables

**Table 1:** Measured variables at the baseline.

Measured Variables	Baseline (Mean ± SD)		*p*-Value
	Day 1	Day 2	
MAS for ankle plantar flexor	2.78 ± 0.80	2.78 ± 0.80	1.000
MAS for hip adductors	2.00 ± 0.55	2.00 ± 0.55	1.000
FTSST (sec)	21.29 ± 2.39	19.77 ± 2.00	0.124
10 MWT (sec)	31.00 ± 11.42	30.78 ± 11.11	0.957
Step width (cm)	18.35 ± 3.61	18.33 ± 3.66	0.987
Stride (cm)	37.65 ± 18.77	40.52 ± 17.81	0.687
Step length (cm)	24.95 ± 8.81	25.03 ± 8.80	0.980

Data are presented as mean ± SD, MAS; Modified Ashworth scale, FTSST; five times sit-to-stand test, 10 MWT; 10-m walk test, the significance level was set at *p* < 0.05.

**Table 2:** Measured variables during the short- and long-term management programs versus the baseline data.

Assessed Parameters	Short-Term Management Assessments	*p*-Value	Long-Term Management Assessments			
			Without FES	*p*-Value	With FES	*p*-Value
MAS for ankle plantar flexor	1.71 ± 0.72	0.001	1.92 ± 0.91	0.006	0.85 ± 0.77	0.001
MAS for hip adductors	1.00 ± 0.55	0.001	1.00 ± 0.55	0.001	0.35 ± 0.63	0.001
FTSST (sec)	15.63 ± 2.69	0.001	17.59 ± 3.33	0.001	13.41 ± 2.20	0.001
10 MWT (sec)	22.48 ± 0.21	0.039	21.49 ± 10.06	0.021	20.58 ± 9.97	0.012
Step width (cm)	21.85 ± 3.41	0.012	22.22 ± 3.57	0.006	23.73 ± 3.73	0.001
Stride (cm)	52.20 ± 9.07	0.044	53.35 ± 19.16	0.030	55.31 ± 19.00	0.028
Step length (cm)	32.67 ± 9.16	0.027	33.67 ± 9.16	0.013	35.45 ± 9.08	0.003

Data are presented as mean ± SD, MAS; Modified Ashworth scale, FTSST; five times sit-to-stand test, 10 MWT; 10-m walk test, repeated measures one-way ANOVA tests between baseline versus short-term and long-term management programs was performed, the significance level was set at *p* < 0.05.

## Data Availability

The original contributions presented in this study are included in the article. Further inquiries can be directed to the corresponding author(s).
